# The crosstalk between STAT3 and microRNA in cardiac diseases and protection

**DOI:** 10.3389/fcvm.2022.986423

**Published:** 2022-09-06

**Authors:** Lan Wu, Zhizheng Li, Yanfei Li

**Affiliations:** ^1^Affiliated Zhoupu Hospital and Shanghai Key Laboratory of Molecular Imaging, Shanghai University of Medicine and Health Sciences, Shanghai, China; ^2^School of Basic Medical Sciences, Shanghai University of Medicine and Health Sciences, Shanghai, China; ^3^School of Medical Technology, Shanghai University of Medicine and Health Sciences, Shanghai, China

**Keywords:** signal transducer and activator of transcription 3, microRNA, crosstalk, cardioprotection, cardiac diseases

## Abstract

Signal transducer and activator of transcription 3 (STAT3), an important transcription factor and signaling molecule, play an important role in cardiac disease and protection. As a transcription factor, STAT3 upregulates anti-oxidative and anti-apoptotic genes but suppresses anti-inflammatory and anti-fibrotic genes in cardiac disease and protection. As a signaling molecule, STAT3 is the downstream or upstream of other molecules for signaling transduction, also activated in cardiac disease and protection. MicroRNAs (miRNAs) are endogenous short non-coding RNAs that regulate mRNA expression at the transcriptional level and prevent protein translation. Recently, STAT3 is reported to be not only the target of miRNA but also the inhibitor or inducer of miRNA to modify the mRNA expression profiles in cardiomyocytes resulting in different effects on cardiac disease and protection. We summarize the current knowledge on STAT3 regulation of individual miRNAs and the modulation of STAT3 by miRNAs in cardiac diseases and protection.

## Introduction

Cardiac diseases are the leading causes of morbidity and mortality in the world (1). A remarkable amount of studies have shown the multifaceted roles of signal transducer and activator of transcription 3 (STAT3) and microRNAs (miRNAs) in cardiac disease and protection. STAT3 is a key transcript factor and signaling protein of many cytokines and growth factors (2). MiRNAs are emerging as important gene expression regulators of physiological and pathological process (3).

STAT3 participates in protection against myocardial infarction (MI), cardiac ischemia/reperfusion (I/R) injury, and peripartum cardiomyopathy (PPCM) by reducing apoptosis, increasing the expression of survival proteins, protecting mitochondrial respiration and metabolism, and delaying mitochondrial permeability transition pore (mPTP) opening. On the other hand, STAT3 participates in pathological mechanisms contributing to cardiac hypertrophy, cardiac fibrosis, and septic cardiomyopathy by promoting the growth of cardiomyocytes, collagen synthesis, and the progress of inflammation (2). MiRNAs are also involved in cardioprotection and cardiac pathology by working as counterparts of transcription factors acting at the post-transcriptional level where they target mostly the 3' untranslated region (UTR) of the gene (3).

Although there is huge progress in the function of STAT3 and miRNAs in cardiac disease and protection, the information on the crosstalk between STAT3 and miRNA is scarce. This review presents that STAT3 serves as the target of miRNAs and the upstream regulators of miRNAs that manage their transcriptional control in different cardiac diseases and protection. Most of the findings on STAT3/miRNAs crosstalk have been published over the past few years, and this is the first review specifically focused on this subject to our knowledge.

## Overview of STAT3 and miRNA in heart

### Cardiac STAT3

STAT3 is a DNA-binding protein composed of several domains. STAT3 exists in two main isoforms, full-length STAT3a, and truncated STAT3b generated by alternative splicing. STAT3a protein has six domains: transactivation domain (TAD), DNA-binding domain (DBD), linker domain (LD), Src homology 2 (SH2), N-terminal domain (NTD), and coiled-coil domain (CCD), whereas TAD is absent in STAT3b. These six domains perform their respective functions in functional regulation, which are as follows: Amino acid residues, tyrosine 705 and serine 727, in TAD are phosphorylation sites, which are the main pathway of STAT3 activation; DBD is used to bind to specific DNA sequences; LD can connect DBD and SH2 domains; SH2 affects the formation of STAT3 dimerization; NTD affects STAT3 dimerization into the nucleus and DNA binding; and CCD is mainly responsible for recruiting STAT3 to bind to receptors (4).

Janus 2 (JAK2)/STAT3 is an evolutionarily conserved signal transduction pathway that is widely involved in the regulation of inflammation, apoptosis, cell cycle, and development, and is involved in many myocardial diseases including myocardial infarction, oxidative damage, myocarditis, myocardial hypertrophy, and ventricular remodeling. It is not only an indispensable stress response pathway but also plays an important role in cardioprotection such as ischemia preconditioning (IPC) and ischemia postconditioning (IPoC). Activation of cell surface tyrosine kinase receptors promoted phosphorylation of the tyrosine 705 site of STAT3 to form dimers and enter the nucleus for DNA binding to upregulate gene transcription, such as anti-apoptotic (B cell lymphoma/leukemia-2 (Bcl-2), B cell lymphoma/leukemia-xl (Bcl-xl), myeloid cell leukemia-1 (Mcl-1), FLICE [Fas-associated death domain-like IL-lbeta-converting enzyme-inhibitory protein (c-FLIP)], anti-oxidative (MnSOD and Metallothionein MT1/MT2), and cardioprotective [Cyclooxygenase-2 (COX-2) and heme oxygenase (HO-1)] proteins (5). While phosphorylation at serine 727 of STAT3 by serine/threonine kinases was also demonstrated to not only be required for further transcriptional activity but also regulated mitochondrial respiration in mitochondria and promoted oxidative phosphorylation through controlling electron transport chain (ETC) activity and adenosine 5′-triphosphate (ATP) production. Besides phosphorylation, the gene transcription was also regulated by acetylation of lysine residues within its NTD and SH2 of STAT3 through p300 (6).

### Cardiac MiRNA

MiRNA is a class of non-coding single-stranded RNA molecules of ~22–24 nucleotides(nt) in length encoded by endogenous genes. Most miRNA genes exist in the genome in the form of a single copy, multiple copies, or gene clusters. Pri-miRNA is the most primitive form of miRNA and its length is about 300–1,000 nt. Pri-miRNA becomes a miRNA precursor (pre-miRNA) after processing and its length is about 70–90 nt. After pre-miRNA is digested by the Dicer enzyme, it becomes mature miRNA with a length of about 20–24 nt. The mature miRNA loads to form an RNA-induced silencing complex (RISC) and binds to its target mRNA, preventing its translation into a protein (7).

Some miRNAs are highly abundant in cardiomyocytes for preserving cardiac function against cardiac hypertrophy, such as miR-133 and miR-1, while other miRNAs should be inhibited in cardiomyocytes for improving cardiac contractility, such as miR-208. Some miRNAs promote cardiomyocyte proliferation as an alternative to cell therapy for myocardial infarction, such as miR-199a and miR-590, while other miRNAs lead to cardiomyocyte apoptosis and telomere shortening and aggravate myocardial infarction injury, such as miR-34a. Some miRNAs prevent cardiac fibrosis, such as miR-133, while other miRNAs contribute to cardiac fibrosis, such as miR-21 (8).

### STAT3-MiRNAs circuits

Recently, STAT3 is reported to be not only the target of miRNA but also the inhibitor or inducer of miRNA to modify the mRNA expression profiles in cardiomyocytes resulting in different effects on cardiac diseases and protection (Table 1). A better understanding of the complex regulatory networks between STAT3 and miRNAs may lead to novel specific therapeutic approaches in various cardiac disease settings.

**Table 1:** STAT3-miRNAs circuits in different cardiac diseases and protection.

**Cardiac diseases**	**STAT3-miRNAs crosstalk**					
	**STAT3 is the direct target of miRNA**	**STAT3 initiates the transcription of miRNA**	**STAT3 inhibits the expression of miRNA**	**STAT3 is the signaling molecule of the target of miRNA**	**STAT3 downregulated miRNA through lncRNA**	**STAT3 is only the downstream molecule of miRNA**
Myocardial infarction (MI)	miR-124	miR-17/20a	miR-155-5p	miR-216a		miR-503
	miR-17-5p	miR-211	miR-34b	miR-135a-5p		miR-29a
	miR-21-5p	miR-199a-5p	miR-337	miR-181a-5p		
	miR-874			miR-106b-5p		
				miR-22		
				miR-15b		
				miR-101a-3p		
				miR-421		
				miR-200c-3p		
Hypertrophic cardiomyopathy (HC)	miR-625-5p		miR-16		miR-361-5p	
	miR-17-5p					
Dilated cardiomyopathy (DCM)	Some miRNA?		miR-199a	miR-148a		
				miR-21		
				miR-320		
Peripartum cardiomyopathy (PPCM)			miR-7a-5p			
			miR-146a			
Septic cardiomyopathy (SC)	miR-125b					
	miR-223					
Drug-induced cardiomyopathy (DIC)	miR-526-3p	miR-21-5p				
Rheumatic heart disease (RHD)				miR-155-5p		

## STAT3-miRNA crosstalk in myocardial infarction

Myocardial infarction (MI) represents myocardial necrosis due to persistent ischemia and hypoxia due to coronary artery occlusion. STAT3-miRNA crosstalk presents in four mechanisms. First, STAT3 is the direct target of miRNA. MiR-124 and miR-17-5p bind the 3'-UTR of STAT3 mRNA and downregulate its expression to aggravate inflammation, autophagy, apoptosis, and myocardial remodeling in MI (9, 10). MiR-98 targets the STAT3 to reduce Bcl-2 expression and melatonin inhibits miR-98 to enhance the therapeutic efficacy of cardiac progenitor cells for MI (11). MiR-21-5p targets STAT3 to reduce inflammation as a biomarker for MI (12). Second, STAT3 works as a transcription factor binding the promoter of the miRNA gene to initiate the expression of miRNA. STAT3 could bind the promoter region of the miR-17-92 gene to induce miR-17/20a targeting prolyl hydroxylase 3 (PHD3), which attenuates apoptosis in MI (13). STAT3 could directly bind the promoter elements to activate miR-211 expression targeting signal transducer and activator of transcription 5A (STAT5A), which enhanced protection from adverse post-MI remodeling in the therapy of the mesenchymal stem cells transplant (14). Third, STAT3 works as the signaling molecule of other proteins, which is the target of miRNA. Long non-coding RNA NR_045363 sponged miR-216a to increase JAK2 and thus activated STAT3 to promote cell proliferation and cardiac repair in MI. Fourth, STAT3 is only the downstream molecule of miRNA (15). Chemokine (C-X-C Motif) ligand 12 (CXCL12) is a target gene of miR-135a-5p and thus miR-135a-5p inhibited the activation of the JAK2/STAT3 signaling pathway to reduce inflammatory reaction and myocardial cell apoptosis in MI (16). In conclusion, a decrease in STAT3 activation by miRNA and a decrease in pro-apoptotic protein by STAT3-miRNA play dual roles in MI.

### STAT3-miRNAs circuits in cardiac ischemia/hypoxia injury

Ischemia/hypoxia is the cause of MI, during which apoptotic and necrotic cell death occurs. STAT3-miRNA crosstalk presents in three mechanisms. First, STAT3 works as a transcription factor binding the promoter of the miRNA gene to initiate the expression of miRNA. Activated STAT3 could bind to the promoter region of miR-199a-2 gene, pri-miR-199a-2, a precursor of miR-199a-5p, of which targets are endoplasmic reticulum stress (ERS)-related activating transcription factor 6 (ATF6) and 78 kDa glucose-regulated protein (GRP78) to prevent unfolded protein response for cardioprotection in hypoxic cardiomyocytes (17). Second, STAT3 works as the inhibitor of miRNA transcription to result in the downregulation of miRNA. Activated STAT3 also inhibited miR-155-5p transcription, of which target is endothelial PAS domain protein 1 (EPAS1) to promote the activation of downstream vascular endothelial growth factor (VEGF), basic fibroblast growth factor (bFGF), and interleukin 6 (IL-6) to induce cardioprotection in ischemic cardiomyocytes (18). Third, STAT3 works as the signaling molecule of other proteins, which is the target of miRNA. Long non-coding RNA myocardial infarction-associated transcript (LncRNA MIAT) sponged miR-181a-5p to enhance the expression of JAK2 and thus increased the JAK2/STAT3 signaling pathway to induce inflammation and apoptosis in oxygen-glucose deprivation-induced cardiomyocyte (19). LncRNA HOX transcript antisense RNA (HOTAIR) sponged miR-106b-5p to activate AKT and STAT3 pathways to alleviate H_2_O_2_-stimulated oxidative stress in H9c2 cells (20). LncRNA CAMK2D-associated transcript (C2dat1) sponged miR-22 to upregulate VEGF, which further enhanced the activation of JAK/STAT3 pathways and alleviated hypoxia injury in H9c2 cells (21). Overall, STAT3-miRNA crosstalk plays pivotal role in regulating the protective or injured cardiac response during ischemia/hypoxia injury.

### STAT3-miRNAs circuits in cardiac ischemia/reperfusion injury

Ischemia/reperfusion (I/R) injury is a pathological state caused by an initial low supply of blood to a specific area (ischemia), followed by the restoration of perfusion and reoxygenation (reperfusion). I/R is another cause of MI injury in treatment. STAT3-miRNA crosstalk presents in four mechanisms. First, STAT3 is the direct target of miRNA. MiR-874 and miR-17-5p bind the 3'-UTR of STAT3 mRNA and downregulate its expression to induce cardiomyocyte apoptosis in I/R (22, 23). HOTAIR adsorbed miR-17-5p to promote cardioprotection against I/R injury (24). Second, STAT3 works as the inhibitor of miRNA to result in the downregulation of miRNA. Activated STAT3 also inhibited miR-34b and miR-337 expression against cardiac IR injury; however, the target proteins of miR-34b and miR-337 were unknown (25). Third, STAT3 works as the signaling molecule of other proteins, which is the target of miRNA. MiR-15b and miR-101a-3p bind the 3'-UTR of JAK2 mRNA to inhibit the JAK2/STAT3 signaling pathway, which leads to cardiomyocyte apoptosis aggravating myocardial I/R injury (26, 27). MiR-421 directly targets toll-like receptor 4 (TLR4), which is the upstream of the JAK2/STAT3 signaling pathway in inflammation in I/R injury (28). MiR-200c-3p directly targets at adiponectin receptor 2 (AdipoR2), which activated the STAT3 signaling pathway to suppress apoptosis in I/R (29). Fourth, STAT3 is the indirect target of miRNA to reduce the phosphorylation of STAT3. MiR-503 not only directly targets at phosphatidylinositol-3-kinase (PI3K) and Bcl-2 but also reduces the phosphorylation of STAT3 leading to myocyte apoptosis in I/R injury (30). MiR-29a directly targets at and inhibits follistatin-like 1 (FSTL1) secretion and promotes myocyte apoptosis by suppressing the JAK2/STAT3 pathway in hypoxia-reoxygenation injury (31). Taken together, the decrease in STAT3 activation by miRNA would lead to apoptosis in I/R injury.

## STAT3-miRNA crosstalk in hypertrophic cardiomyopathy

Hypertrophic cardiomyopathy (HC) is an unexplained cardiac disease characterized by asymmetric hypertrophy of the ventricular wall, often invading the interventricular septum, smaller ventricular chamber, obstruction of left ventricular blood filling, and decreased left ventricular diastolic compliance. STAT3-miRNA crosstalk presents in three mechanisms. First, STAT3 is the direct target of miRNA. MiR-625-5p and miR-17-5p could bind the 3'-UTR of STAT3 mRNA and downregulated its expression to attenuate cardiac hypertrophy (32, 33). Second, STAT3 down regulated miRNA through lncRNA. STAT3 could bind to the promoter region of long non-coding RNA maternally expressed gene 3 (MEG3) to upregulate MEG3, which acts as a ceRNA competitively binding with miR-361-5p. MiR-361-5p directly targets recombinant histone deacetylase 9 (HDAC9), and thus STAT3 promotes cardiac hypertrophy (34). Third, STAT3 works as the inhibitor of miRNA transcription to result in the downregulation of miRNA. STAT3 inhibited the miR-16 targeting at cyclins D1, D2, and E1 and provoke cardiomyocyte hypertrophy (35). Overall, the above findings indicate that the role of STAT3-miRNA crosstalk in hypertrophy is relatively clear.

## STAT3-miRNA crosstalk in dilated cardiomyopathy

Dilated cardiomyopathy (DCM) manifests as left or right ventricle or bilateral ventricular enlargement with ventricular systolic hypofunction, with or without congestive heart failure. STAT3-miRNA crosstalk presents in two mechanisms. First, STAT3 works as the signaling molecule of other proteins, which is the target of miRNA. MiR-148a targets the cytokine co-receptor glycoprotein 130 (gp130), which is the positive upstream of STAT3, and suppresses ventricular dilation (36). Second, STAT3 is the direct target of miRNA. Some miRNA predicted to target STAT3, which is an inhibitor of ferroptosis in DCM (37). In summary, STAT3-miRNA protects the heart against DCM.

### STAT3-miRNAs circuits in cardiac fibrosis

Progressive fibrosis of the endocardial and subendocardial myocardium results in decreased ventricular wall compliance and heart stenosis, which is an important pathology of DCM. STAT3-miRNA crosstalk presents in only one mechanism. STAT3 works as the signaling molecule of other proteins, which is the target of miRNA. MiR-21 targets tumor suppressor cell adhesion molecule 1 (CADM1), which is the negative upstream of STAT3, and enhances cardiac fibrosis (38). MiR-320 targets Krüppel-like factor 9 (KLF9), which is also the negative upstream of STAT3, and accelerates cardiac fibrosis (39). All the evidence indicates that STAT3 is an important contributor to cardiac fibrosis, whereas miRNA can deregulate STAT3.

### STAT3-miRNAs circuits in cardiac structure

Promoting myocardial cell proliferation and maintaining myocardial cytoskeletal structure is an important treatment of DCM. STAT3-miRNA crosstalk presents in only one mechanism. STAT3 works as the inhibitor of miRNA transcription to result in the downregulation of miRNA. STAT3 suppresses promoter activity of the miR-199a-2 gene in cardiomyocytes. MiR-199a suppressed the expression of ubiquitin-conjugating enzymes e2i (Ube2i) and ubiquitin-conjugating enzyme e2g1 (Ube2g1) in cardiomyocytes. The two proteins are important for cardiomyocyte ultrastructure and expression of sarcomeric myosin heavy chain (40). STAT3-miRNA crosstalk protects the myocardial structure.

## STAT3-miRNA crosstalk in peripartum cardiomyopathy

Peripartum cardiomyopathy (PPCM) is a rare heart disease that develops in women during the last month of pregnancy or within 5 months of delivery. Its typical features are apoptosis, inflammation, autoimmune processes, and impaired cardiac microvasculature. STAT3-miRNA crosstalk presents in only one mechanism. STAT3 works as the inhibitor of miRNA transcription to result in the downregulation of miRNA. STAT3 plays a protective role in PPCM by inhibiting three miRNAs: miR-7a-5p, which suppressed glucose transporter-4 (GLUT4) levels, miR-199a-5p, which suppressed expression of the cardioprotective receptor for neuregulin (ErbB), and miR-146a, which suppressed ErbB4, N rat sarcoma (Nras), Notch homolog 1 (Notch1), and Interleukin 1 Receptor Associated Kinase 1 (Irak1) (41). Evidence showed that STAT3 inhibits miRNA and protects against PPCM.

## STAT3-miRNA crosstalk in septic cardiomyopathy

Septic cardiomyopathy (SC) refers to the subsequent cardiomyopathy with the aggravation of the primary disease when severe infection, severe pancreatitis, and sepsis occur. STAT3-miRNA crosstalk presents in only one mechanism. STAT3 is the direct target of miRNA. MiR-125b is directly targeted at STAT3 mRNA and STAT3 bound with high mobility group box 1 (HMGB1) promoter to enhance autophagy in SC (42). MiR-21 directly targeted STAT3 and protein phosphatase 1 regulatory subunit 3A (PPP1R3A) to result in inflammation in SC. MiR-223 directly targeted at STAT3 and semaphorin 3A confer protection against cecal ligation and puncture (CLP)-triggered cardiac dysfunction, apoptosis, and inflammatory response (43). Taken together, a decrease in STAT3 by miRNA promotes inflammation in SC.

## STAT3-miRNA crosstalk in drug-induced cardiomyopathy

Drug-induced cardiomyopathy (DIC) refers to direct or indirect cardiotoxicity of the drug. STAT3-miRNA crosstalk presents in two mechanisms. First, STAT3 is the direct target of miRNA. MiR-526-3p is targeted at STAT3 to reduce vascular endothelial growth factor A (VEGFA) transcription to aggravate DIC (44). Second, STAT3 works as a transcription factor binding the promoter of the miRNA gene to initiate the expression of miRNA. STAT3 upregulated miR-21-5p in the heart to induce inflammation upon acute drug-induced cardiac injury (45). Thus, the role of STAT3-miRNA crosstalk in DIC is still controversial and requires further investigation.

## STAT3-miRNA crosstalk in rheumatic heart disease

Rheumatic heart disease (RHD) refers to heart valve disease caused by rheumatic fever activity involving the heart valve. STAT3-miRNA crosstalk presents in only one mechanism. STAT3 works as the signaling molecule of other proteins, which is the target of miRNA. MiR-155-5p targeted at suppressor of cytokine signaling 1 (SOCS1), which negatively regulated STAT3 phosphorylation in RHD (46). This evidence suggests that STAT3-miRNA crosstalk in RHD is relatively clear.

## Conclusion and perspectives

**Figure 1:**
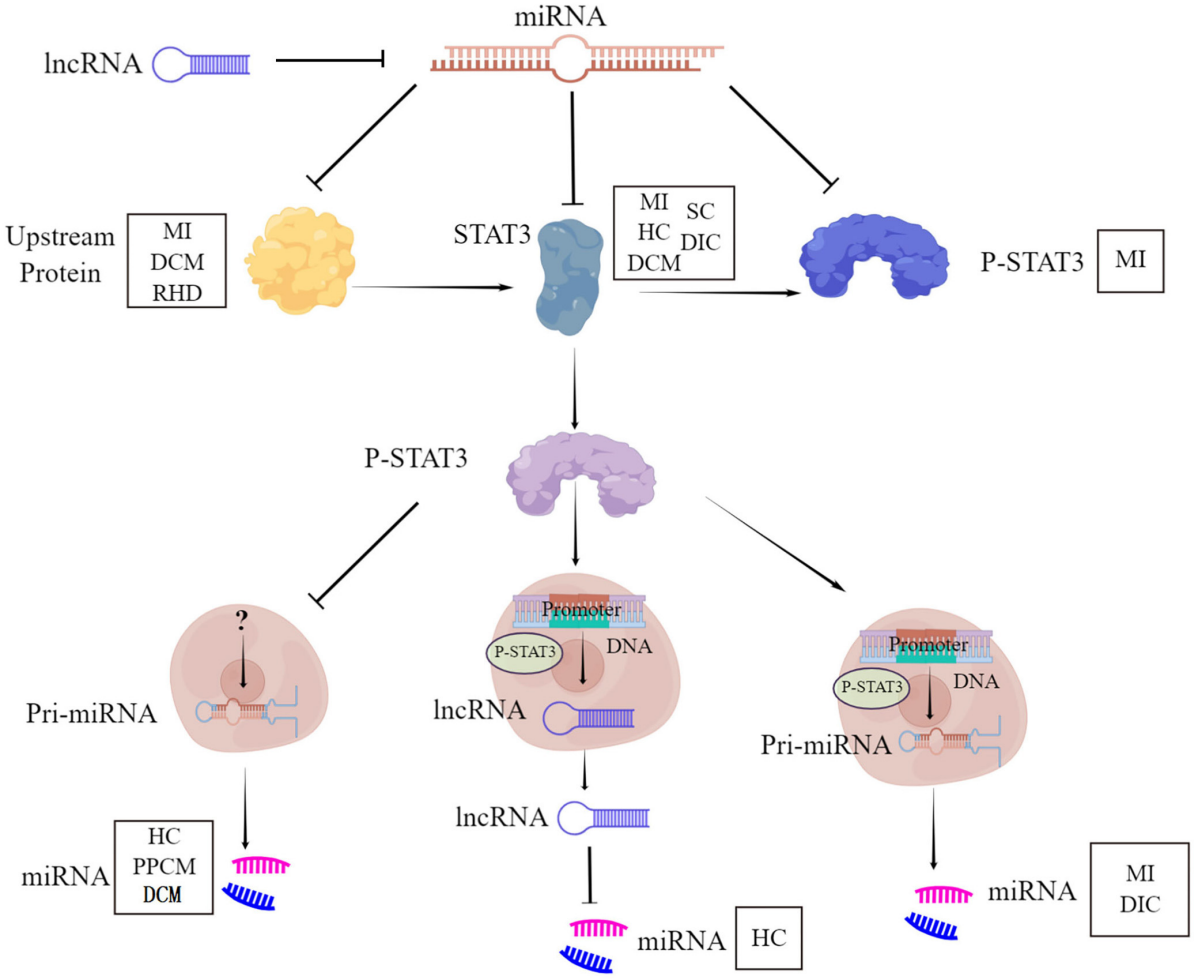
STAT3-miRNA crosstalk. STAT3, Signal Transducer and Activator of Transcription 3; P-STAT3, phosphorylation of STAT3; miRNA, MicroRNA; lncRNA, Long non-coding RNA; MI, Myocardial infarction; DCM, Dilated cardiomyopathy; RHD, Rheumatic heart disease; HC, Hypertrophic cardiomyopathy; SC, Septic cardiomyopathy; DIC, Drug-induced cardiomyopathy; PPCM, Peripartum cardiomyopathy.

STAT3-miRNA crosstalk represents a crucial circuit for the maintenance of heart function, under physiological and pathological conditions. The cardiac diseases include myocardial infarction, hypertrophic cardiomyopathy, dilated cardiomyopathy, peripartum cardiomyopathy, septic cardiomyopathy, drug-induced cardiomyopathy, and rheumatic cardiomyopathy. This involvement of STAT3-miRNA crosstalk is mediated through six aspects (Figure 1). First, STAT3 is the direct target of miRNA. Second, STAT3 works as a transcription factor binding the promoter of the miRNA gene to initiate the expression of miRNA. Third, STAT3 works as the inhibitor of miRNA transcription to result in the downregulation of miRNA. Fourth, STAT3 works as the signaling molecule of other proteins, which is the target of miRNA. Fifth, STAT3 is only the downstream molecule of miRNA. Sixth, STAT3 down regulated miRNA through lncRNA. Whether there are other cardiac diseases and other regulatory mechanisms need to be deciphered for a better understanding of STAT3-miRNA crosstalk.

Cancer-induced cardiac cachexia is an insidious cardiac disease with a dramatic impact on a patient's quality of life and survival (47). Tumor-derived cytokines on cardiac muscle increased protein degradation, enhanced reactive oxygen species (ROS) production, re-express fetal genes, and impaired fatty acid oxidation. The mechanisms of cancer-induced cardiac cachexia included inflammation, abnormal metabolism, atrophy, and proteolysis. Combined effects of inflammation and oxidative stress stimulate atrophy, reduction in oxidative capacity, and mitochondrial dysfunction. Activation of nuclear factor kappa-B (NF-kB) increases ubiquitin/proteasome-dependent proteolysis and contributes to the dysregulation of oxidative metabolism (48). STAT3 inhibited the NF-kB signaling pathway and reduced inflammatory responses in the heart (49). This suggests that STAT3 may contribute to cardioprotection against cancer-induced cardiac cachexia. However, STAT3-miRNA crosstalk has not been reported in cancer-induced cardiac cachexia. Thus, this is a promising study point worthy of deep exploration.

In therapy, understanding the underlying mechanism of the pathology is the first step in discovering preventative or therapeutic options and drugs for this pathology. Since STAT3-miRNA crosstalk is involved in a wide range of cardiac diseases and protection, understanding the mechanisms of this involvement paves the way toward finding therapeutic strategies for either the regulation of STAT3 or the change of miRNA. This review also highlights several targets of STAT3-miRNA crosstalk for therapeutic approaches. Especially in cancer-induced cardiac cachexia, a new and important study field of cardiomyopathy, the roles of STAT3-miRNA crosstalk remain unknown and deserve deep investigation. The pharmacological or genetic intervention of STAT3 or miRNA which influence the downstream targets may be good therapeutic approach for different cardiac diseases.

## Author contributions

LW wrote the manuscript and created the figure and table. ZL helped drawing the figure. YL helped editing the text. All authors contributed to the article and approved the submitted version.

## Funding

This work was supported by grants from the Special Clinical Research Project of Shanghai Municipal Health Commission (20204Y0378), the Academic Mentorship for Scientific Research Cadre Project of Shanghai University of Medicine and Health Sciences (E3-0200-21-201012-2), and the Construction project of Shanghai Key Laboratory of Molecular Imaging (18DZ2260400).

## Conflict of interest

The authors declare that the research was conducted in the absence of any commercial or financial relationships that could be construed as a potential conflict of interest.

## Publisher's note

All claims expressed in this article are solely those of the authors and do not necessarily represent those of their affiliated organizations, or those of the publisher, the editors and the reviewers. Any product that may be evaluated in this article, or claim that may be made by its manufacturer, is not guaranteed or endorsed by the publisher.

## References

[1] Mahmood SS Levy D Vasan RS Wang TJ. The Framingham Heart Study and the epidemiology of cardiovascular disease: a historical perspective. Lancet. (2014) 383:999–1008. 10.1016/S0140-6736(13)61752-3 24084292 PMC4159698

[2] Kurdi M Zgheib C Booz GW. Recent developments on the crosstalk between STAT3 and inflammation in heart function and disease. Front Immunol. (2018) 9:3029. 10.3389/fimmu.2018.03029 30619368 PMC6305745

[3] Bernardo BC Ooi JY Lin RC McMullen JR. miRNA therapeutics: a new class of drugs with potential therapeutic applications in the heart. Future Med Chem. (2015) 7:1771–92. 10.4155/fmc.15.107 26399457

[4] Qi Q-R Yang Z-M. Regulation and function of signal transducer and activator of transcription 3. World J Biol Chem. (2014) 5:231–9. 10.4331/wjbc.v5.i2.231 24921012 PMC4050116

[5] Zouein FA Altara R Chen Q Lesnefsky EJ Kurdi M Booz GW. Pivotal importance of STAT3 in protecting the heart from acute and chronic stress: new advancement and unresolved issues. Front Cardiovasc Med. (2015) 2:36. 10.3389/fcvm.2015.00036 26664907 PMC4671345

[6] Harhous Z Booz GW Ovize M Bidaux G Kurdi M. An update on the multifaceted roles of STAT3 in the heart. Front Cardiovasc Med. (2019) 6:150. 10.3389/fcvm.2019.00150 31709266 PMC6823716

[7] Fabian MR Sonenberg N. The mechanics of miRNA-mediated gene silencing: a look under the hood of miRISC. Nat Struct Mol Biol. (2012) 19:586–93. 10.1038/nsmb.2296 22664986

[8] Barwari T Joshi A Mayr M. MicroRNAs in cardiovascular disease. J Am Coll Cardiol. (2016) 68:2577–84. 10.1016/j.jacc.2016.09.945 27931616

[9] He F Liu H Guo J Yang D Yu Y Yu J. Inhibition of MicroRNA-124 reduces cardiomyocyte apoptosis following myocardial infarction via targeting STAT3. Cell Physiol Biochem. (2018) 51:186–200. 10.1159/000495173 30439699

[10] Chen B Yang Y Wu J Song J Lu J. microRNA-17-5p downregulation inhibits autophagy and myocardial remodelling after myocardial infarction by targeting STAT3. Autoimmunity. (2022) 55:43–51. 10.1080/08916934.2021.1992754 34755577

[11] Ma W He F Ding F Zhang L Huang Q Bi C. Pre-treatment with melatonin enhances therapeutic efficacy of cardiac progenitor cells for myocardial infarction. Cell Physiol Biochem. (2018) 47:1287–98. 10.1159/000490224 29913449

[12] Su J Gao C Wang R Xiao C Yang M. Genes associated with inflammation and the cell cycle may serve as biomarkers for the diagnosis and prognosis of acute myocardial infarction in a Chinese population. Mol Med Rep. (2018) 18:1311–22. 10.3892/mmr.2018.9077 29845217 PMC6072145

[13] Samidurai A Roh SK Prakash M Durrant D Salloum FN Kukreja RC. STAT3-miR-17/20 signalling axis plays a critical role in attenuating myocardial infarction following rapamycin treatment in diabetic mice. Cardiovasc Res. (2020) 116:2103–15. 10.1093/cvr/cvz315 31738412 PMC8463091

[14] Hu X Chen P Wu Y Wang K Xu Y Chen H. MiR-211/STAT5A signaling modulates migration of mesenchymal stem cells to improve its therapeutic efficacy. Stem Cells. (2016) 34:1846–58. 10.1002/stem.2391 27145179 PMC5096301

[15] Wang J Chen X Shen D Ge D Chen J Pei J. A long noncoding RNA NR_045363 controls cardiomyocyte proliferation and cardiac repair. J Mol Cell Cardiol. (2019) 127:105–14. 10.1016/j.yjmcc.2018.12.005 30553885

[16] Guo XY Liu QL Liu W Cheng JX Li ZJ. Effect and mechanism of miR-135a-5p/CXCL12/JAK-STAT axis on inflammatory response after myocardial infarction. Eur Rev Med Pharmacol Sci. (2020) 24:12912–28. 10.26355/eurrev_202012_24195 33378042

[17] Zhou Y Pang B Xiao Y Zhou S He B Zhang F. The protective microRNA-199a-5p-mediated unfolded protein response in hypoxic cardiomyocytes is regulated by STAT3 pathway. J Physiol Biochem. (2019) 75:73–81. 10.1007/s13105-018-0657-6 30426460

[18] Yao Y Zhou J Lu C Sun W Kong W Zhao J. MicroRNA-155-5p/EPAS1/interleukin 6 pathway participated in the protection function of sphingosylphosphorylcholine to ischemic cardiomyocytes. Life Sci. (2021) 264:118692. 10.1016/j.lfs.2020.118692 33130081

[19] Tan JK Ma XF Wang GN Jiang CR Gong HQ Liu H. LncRNA MIAT knockdown alleviates oxygen-glucose deprivation-induced cardiomyocyte injury by regulating JAK2/STAT3 pathway via miR-181a-5p. J Cardiol. (2021) 78:586–97. 10.1016/j.jjcc.2021.08.018 34489160

[20] Xu G Zhang W Wang Z Chen M Shi B. Matrine regulates H_2_O_2_-induced oxidative stress through long non-coding RNA HOTAIR/miR-106b-5p axis via AKT and STAT3 pathways. Biosci Rep. (2020) 40:BSR20192560. 10.1042/BSR20192560 32395744 PMC7251328

[21] Sun H Shi K Xie D Zhang H Yu B. Long noncoding RNA C2dat1 protects H9c2 cells against hypoxia injury by downregulating miR-22. J Cell Physiol. (2019) 234:20623–33. 10.1002/jcp.28667 31004350

[22] Chen PJ Shang AQ Yang JP Wang WW. microRNA-874 inhibition targeting STAT3 protects the heart from ischemia-reperfusion injury by attenuating cardiomyocyte apoptosis in a mouse model. J Cell Physiol. (2019) 234:6182–93. 10.1002/jcp.27398 30370578

[23] Du W Pan Z Chen X Wang L Zhang Y Li S. By targeting Stat3 microRNA-17-5p promotes cardiomyocyte apoptosis in response to ischemia followed by reperfusion. Cell Physiol Biochem. (2014) 34:955–65. 10.1159/000366312 25200830

[24] Chen J Li X Zhao F Hu Y. HOTAIR/miR-17-5p axis is involved in the propofol-mediated cardioprotection against ischemia/reperfusion injury. Clin Interv Aging. (2021) 16:621–32. 10.2147/CIA.S286429 33883889 PMC8055365

[25] Pedretti S Brulhart-Meynet MC Montecucco F Lecour S James RW Frias MA. HDL protects against myocardial ischemia reperfusion injury via miR-34b and miR-337 expression which requires STAT3. PLoS ONE. (2019) 14:e0218432. 10.1371/journal.pone.0218432 31220137 PMC6586303

[26] Wang P Sun J Lv S Xie T Wang X. Apigenin alleviates myocardial reperfusion injury in rats by downregulating miR-15b. Med Sci Monit. (2019) 15;25:2764–76. 10.12659/MSM.912014 30983593 PMC6481235

[27] Liu J Wang J Ning Y Chen F. The inhibition of miR-101a-3p alleviates H/R injury in H9C2 cells by regulating the JAK2/STAT3 pathway. Mol Med Rep. (2020) 21:89–96. 10.3892/mmr.2019.10793 31746349 PMC6896302

[28] Guo LL Guo ML Yao J Weng YQ Zhang XZ. MicroRNA-421 improves ischemia/reperfusion injury via regulation toll-like receptor 4 pathway. J Int Med Res. (2020) 48:300060519871863. 10.1177/0300060519871863 31847632 PMC7607211

[29] Huang L Ding L Yu S Huang X Ren Q. Propofol postconditioning alleviates diabetic myocardial ischemia-reperfusion injury via the miR-200c-3p/AdipoR2/STAT3 signaling pathway. Mol Med Rep. (2022) 25:137. 10.3892/mmr.2022.12653 35211763 PMC8908333

[30] He Y Cai Y Sun T Zhang L Irwin MG Xu A. MicroRNA-503 exacerbates myocardial ischemia/reperfusion injury via inhibiting PI3K/Akt- and STAT3-dependent prosurvival signaling pathways. Oxid Med Cell Longev. (2022) 2022:3449739. 10.1155/2022/3449739 35620576 PMC9130001

[31] Li KS Jiang WP Li QC Zhang HW Bai Y Zhang X. MiR-29a in mesenchymal stem cells inhibits FSTL1 secretion and promotes cardiac myocyte apoptosis in hypoxia-reoxygenation injury. Cardiovasc Pathol. (2020) 46:107180. 10.1016/j.carpath.2019.107180 31945680

[32] Cai K Chen H. MiR-625-5p inhibits cardiac hypertrophy through targeting STAT3 and CaMKII. Hum Gene Ther Clin Dev. (2019) 30:182–91. 10.1089/humc.2019.087 31617427

[33] Shi H Li J Song Q Cheng L Sun H Fan W. Systematic identification and analysis of dysregulated miRNA and transcription factor feed-forward loops in hypertrophic cardiomyopathy. J Cell Mol Med. (2019) 23:306–16. 10.1111/jcmm.13928 30338905 PMC6307764

[34] Zhang J Liang Y Huang X Guo X Liu Y Zhong J. STAT3-induced upregulation of lncRNA MEG3 regulates the growth of cardiac hypertrophy through miR-361-5p/HDAC9 axis. Sci Rep. (2019) 9:460. 10.1038/s41598-018-36369-1 30679521 PMC6346020

[35] Huang S Zou X Zhu JN Fu YH Lin QX Liang YY. Attenuation of microRNA-16 derepresses the cyclins D1, D2 and E1 to provoke cardiomyocyte hypertrophy. J Cell Mol Med. (2015) 19:608–19. 10.1111/jcmm.12445 25583328 PMC4369817

[36] Raso A Dirkx E Philippen LE Fernandez-Celis A De Majo F Sampaio-Pinto V. Therapeutic delivery of miR-148a suppresses ventricular dilation in heart failure. Mol Ther. (2019) 27:584–99. 10.1016/j.ymthe.2018.11.011 30559069 PMC6403487

[37] Wang Z Xia Q Su W Cao M Sun Y Zhang M. Exploring the communal pathogenesis, ferroptosis mechanism, and potential therapeutic targets of dilated cardiomyopathy and hypertrophic cardiomyopathy via a microarray data analysis. Front Cardiovasc Med. (2022) 9:824756. 10.3389/fcvm.2022.824756 35282347 PMC8907834

[38] Cao W Shi P Ge JJ. miR-21 enhances cardiac fibrotic remodeling and fibroblast proliferation via CADM1/STAT3 pathway. BMC Cardiovasc Disord. (2017) 17:88. 10.1186/s12872-017-0520-7 28335740 PMC5364650

[39] Li F Li SS Chen H Zhao JZ Hao J Liu JM. miR-320 accelerates chronic heart failure with cardiac fibrosis through activation of the IL6/STAT3 axis. Aging (Albany NY). (2021) 13:22516–27. 10.18632/aging.203562 34582362 PMC8507257

[40] Haghikia A Missol-Kolka E Tsikas D Venturini L Brundiers S Castoldi M. Signal transducer and activator of transcription 3-mediated regulation of miR-199a-5p links cardiomyocyte and endothelial cell function in the heart: a key role for ubiquitin-conjugating enzymes. Eur Heart J. (2011) 32:1287–97. 10.1093/eurheartj/ehq369 20965886

[41] Stapel B Kohlhaas M Ricke-Hoch M Haghikia A Erschow S Knuuti J. Low STAT3 expression sensitizes to toxic effects of β-adrenergic receptor stimulation in peripartum cardiomyopathy. Eur Heart J. (2017) 38:349–61. 10.1093/eurheartj/ehw086 28201733 PMC5381590

[42] Yu Y Ou-Yang WX Zhang H Jiang T Tang L Tan YF. MiR-125b enhances autophagic flux to improve septic cardiomyopathy via targeting STAT3/HMGB1. Exp Cell Res. (2021) 409:112842. 10.1016/j.yexcr.2021.112842 34563514

[43] Wang X Gu H Qin D Yang L Huang W Essandoh K. Exosomal miR-223 contributes to mesenchymal stem cell-elicited cardioprotection in polymicrobial sepsis. Sci Rep. (2015) 5:13721. 10.1038/srep13721 26348153 PMC4562230

[44] Zhang L Liu L Li X. MiR-526b-3p mediates doxorubicin-induced cardiotoxicity by targeting STAT3 to inactivate VEGFA. Biomed Pharmacother. (2020) 123:109751. 10.1016/j.biopha.2019.109751 31958751

[45] Gryshkova V Fleming A McGhan P De Ron P Fleurance R Valentin JP. miR-21-5p as a potential biomarker of inflammatory infiltration in the heart upon acute drug-induced cardiac injury in rats. Toxicol Lett. (2018) 286:31–8. 10.1016/j.toxlet.2018.01.013 29355689

[46] Chen A Wen J Lu C Lin B Xian S Huang F. Inhibition of miR-155-5p attenuates the valvular damage induced by rheumatic heart disease. Int J Mol Med. (2020) 45:429–40. 10.3892/ijmm.2019.4420 31894293 PMC6984794

[47] Antunes JMM Ferreira RMP Moreira-Gonçalves D. Exercise training as therapy for cancer-induced cardiac cachexia. Trends Mol Med. (2018) 24:709–27. 10.1016/j.molmed.2018.06.002 29980479

[48] Belloum Y Rannou-Bekono F Favier FB. Cancer-induced cardiac cachexia: Pathogenesis and impact of physical activity (Review). Oncol Rep. (2017) 37:2543–52. 10.3892/or.2017.5542 28393216

[49] Li Z Wang C Mao Y Cui J Wang X Dang J. The expression of STAT3 inhibited the NF-kB signalling pathway and reduced inflammatory responses in mice with viral myocarditis. Int Immunopharmacol. (2021) 95:107534. 10.1016/j.intimp.2021.107534 33752081

